# Fullerenes synthesis by combined resistive heating and arc discharge techniques

**DOI:** 10.1186/s40064-016-2994-7

**Published:** 2016-08-11

**Authors:** Pannan Isa Kyesmen, Audu Onoja, Alexander Nwabueze Amah

**Affiliations:** Department of Physics, Faculty of Science, Federal University of Agriculture Makurdi, Makurdi, Nigeria

**Keywords:** Fullerenes synthesis, Arc discharge, Resistive heating, Graphite, Carbon soot

## Abstract

The two main electrode techniques for fullerenes production; the direct arc technique and the resistive heating of graphite rod were employed in this work. One of the electrodes was resistively heated to high temperature and subjected to arc discharge along its length by the second graphite rod. Fullerenes solid were extracted from carbon soot samples collected from an installed arc discharge system using the solvent extraction method. The fullerenes solid obtained from carbon soot collected for 2 min of arc discharge run when one of the electrodes was resistively heated at different voltages all gave higher yields (maximum of 67 % higher, at 150 A arc current and 200 Torr chamber pressure) compared to when no resistive heating was carried out. Scanning electron microscopy and ultraviolet visible spectroscopy analysis carried out on all fullerenes solid indicated the presence of fullerenes. The enhancement of fullerenes production by combined resistive and direct arc techniques shows prospect for possible use at industrial level for large scale production.

## Background

Macroscopic quantities of fullerenes were first produced using the arc discharge method by Kratschmer, Huffman, and coworkers in 1990 (Charlse and Chia-Chun 1994). They first of all carried out resistive heating of graphite rods in helium environment to produce carbon soot containing fullerenes in macroscopic mass before further modifying the technique into an arc-based carbon vaporization process to produce gram quantities of fullerenes (Nirupam et al. 2015). Several works have been done for the macroscopic production of the fullerenes by using other different techniques which include; laser ablation, electron beam evaporation, diffusion flame and ion beam sputtering (Caraman et al. 2006).

Fullerenes have found cutting-edge applications in technology such as the solar cells technology and molecular electronics among many other areas (Yuming and Guang 2014). They are currently one of the most attractive nanomaterials from applications perspective (Krolow et al. 2013). But their wide applications are still undermined by inability to produce very large and inexpensive quantities (Caraman et al. 2006). The search for system designs and ways for synthesizing macroscopic mass of fullerenes still constitute a problem (Churilov 2013). Any study which aimed to contribute towards enhance production of fullerenes will invariably promote advancements in its applications.

The arc discharge technique has been modified in several ways to achieve several advantages. Such modifications include the use of de-mineralized coal electrodes, application of DC power rather than AC, application of low current rather than high AC current, formation of tapered apparatus for gravity-based collection of carbon soot, etc. (Nirupam et al. 2015). This work presents discharge system which used the direct arc and resistive heating technique simultaneously to produce fullerenes carbon nanostructures.

## Materials and methods

### Materials

Stainless steel cylinder, stainless flange, Galvanize Iron sheet, 6 mm graphite rods of 99.99 % purity, high current feed through, AC arc welding transformer(KENDE BX1-180F), vacuum compatible glass plate, Helium gas/cylinder, vacuum pump, stiff brush, a long spatula, Whatman-2 filter paper, toluene solution and diethyl ether.

### Methods

#### Experimental procedure

**Fig. 1:**
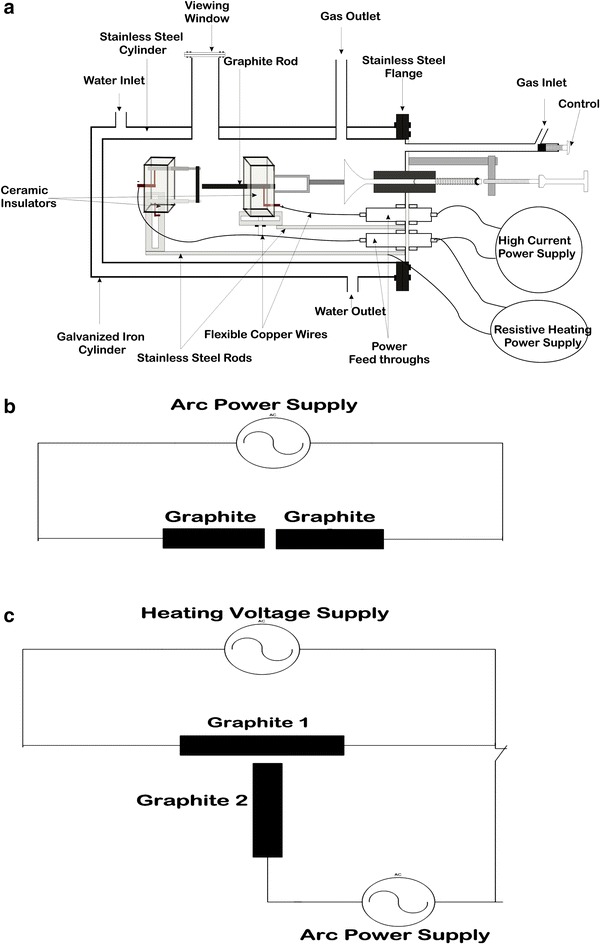
**a** Arc discharge system for fullerenes synthesis, **b** basic circuit diagram for direct arc discharge and **c** the basic circuit diagram that allows for resistive heating of one of the electrodes

The experimental setup in Fig. 1a consists of a double wall, cylindrical chamber with the inner chamber made up of stainless steel. Between the two walls is flowing water which provides cooling for the stainless chamber. The two 6 mm electrodes where first setup in the stainless chamber horizontally as shown in Fig. 1b and secondly in a perpendicular manner as shown in Fig. 1c. One of the electrodes was made movable and guided by a vacuum compatible mechanical system to ensure a constant distance between the electrodes during arc discharge.

First, the two electrodes in the stainless steel chamber were setup as represented in Fig. 1b and made to be 1–2 mm apart. Air inside the stainless steel chamber was pumped out of the chamber using a vacuum pump to a pressure of ≤10^−3^ Torr. Helium gas was then introduced into the chamber at a pressure of 200 Torr. Current of 150 A was supplied to the electrodes using the Arc Welding Transformer power supply. The mechanical system was used to push the movable electrode such that the two electrodes were just touching each other and then released. This initiated an arc discharge between the electrodes which resulted to the vaporization of the graphite electrodes. The arc discharge was made to run for about 2 min. While the arc discharge occurs in the chamber, water was made to circulate round between the walls of the stainless steel chamber cylinder through the water inlet and outlet channels to provide cooling for the chamber.

The arc discharge was made to run for about 2 min. While the arc discharge occurs in the chamber, water was made to circulate round between the walls of the stainless steel chamber cylinder through the water inlet and outlet channels to provide cooling for the chamber. During the arc discharge, part of the carbon rods vapourized and deposited carbon soot on the water cooled wall surface of the stainless steel chamber. The arc discharge that took place inside the stainless chamber was viewed through the viewing window by the aid of a camera. This helped in the control of the movable electrode in the chamber through the mechanical feedthrough.

Secondly, in another procedure, the discharge system was setup as shown in Fig. 1a–c to allow for resistive heating (RH) of one of the electrodes. The same experimental procedure above was repeated except that one of the electrodes was resistively heated at 4.5, 7.5 and 11 V while initiating and maintaining the arc discharge under same arc current of 150 A and chamber pressure of 200 Torr.

#### Soot collection

The helium gas in the chamber was pumped out for about 10 min after the discharge system was allowed to cool down for about 10 min. After this, the flange that carried all the components in the stainless steel chamber was unbolted and removed. The soot deposited on the walls of the stainless steel chamber was scrapped off using a stiff brush and a long spatula and collected into an empty clean container.

#### Solvent extraction of fullerenes

About 250 mg each of the carbon soot that was collected at various system operating conditions of arc discharge current, chamber pressure and resistive heating voltage were mixed with a 100 mg of boiling toluene (fullerenes are soluble in toluene) and stirred for 4 h. The mixtures were then filtered using a Whatman-2 filter paper to obtain fullerenes-toluene solution. Re-crystallization of fullerenes from the toluene solution was carried out by concentrating each of the mixtures using a water bath. The concentrates were then allowed to evaporate at room temperature and brown crystals of fullerenes were collected. The crystals were washed with diethyl ether to remove any hydrocarbon that might be present.

#### Fullerenes characterization

All fullerenes solid extracted from the extraction procedure in “Solvent extraction of fullerenes” section were analyzed using scanning electron microscope (SEM) and ultraviolet–visible (UV–Vis) spectroscopy (in the visible region) techniques. The SEM analysis was done using Phenom ProX desktop scanning electron microscope while the UV–Vis analysis was done using Spectro UV–Vis (UV-2500 series) spectrophotometer respectively.

## Results

### Carbon soot collection

Table 1 shows carbon soot yields and the relative amount of fullerenes extracted using the Arc discharge and Arc discharge/resistive heating methods (at 4.5, 7.5 and 11 V) while maintaining the arc discharge under same arc current of 150 A and chamber pressure of 200 Torr.

**Table 1:** Carbon soot collection and fullerenes yield at all arc discharge system operation conditions

Arc discharge system operating condition of Arc current and chamber pressure	Carbon soot collected for 2 min of arc discharge (mg)	Fullerenes solid in carbon soot collected for 2 min of arc discharge (mg)	Fullerenes Solid Extracted from 250 mg of carbon soot (mg)	Percentage of fullerenes solid extracted from carbon soot (%)
(150 A, 200 Torr)	43.67	2.62	15	6.00
(150 A, 200 Torr, RH4.5 V)	93.50	3.37	9	3.60
(150 A, 200 Torr, RH7.5 V)	108.44	3.04	7	2.80
(150 A, 200 Torr, RH11 V)	110.00	4.40	10	4.00

### Fullerenes extraction

**Fig. 2:**
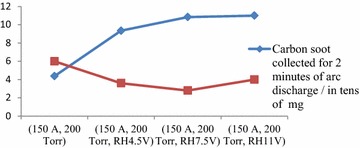
Graph of carbon soot collected for 2 min of arc discharge run/percentage of fullerenes solid present in carbon soot collected at different arc discharge system operating conditions

**Fig. 3:**
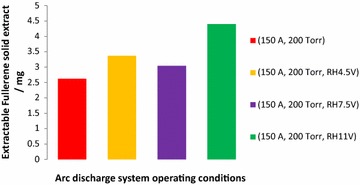
Chart of fullerenes solid extracted from carbon soot collected for 2 min of arc discharge run at different discharge system operating conditions

Table 1 also shows the mass of fullerenes solid extracted from carbon soot at discharge system operating condition of 150 A of arc discharge current and 200 Torr of chamber pressure at no resistive heating, 4.5, 7.5 and 11 V resistive heating voltages. And Fig. 2 shows the graph of carbon soot collected for 2 min of arc discharge run/percentage of fullerenes solid present in carbon soot collected at different arc discharge system operating conditions. Also Fig. 3 shows the chart of fullerenes solid extracted from carbon soot collected for 2 min of arc discharge run at different discharge system operating conditions.

### Fullerenes characterization

#### Scanning electron microscopy (SEM)

**Fig. 4:**
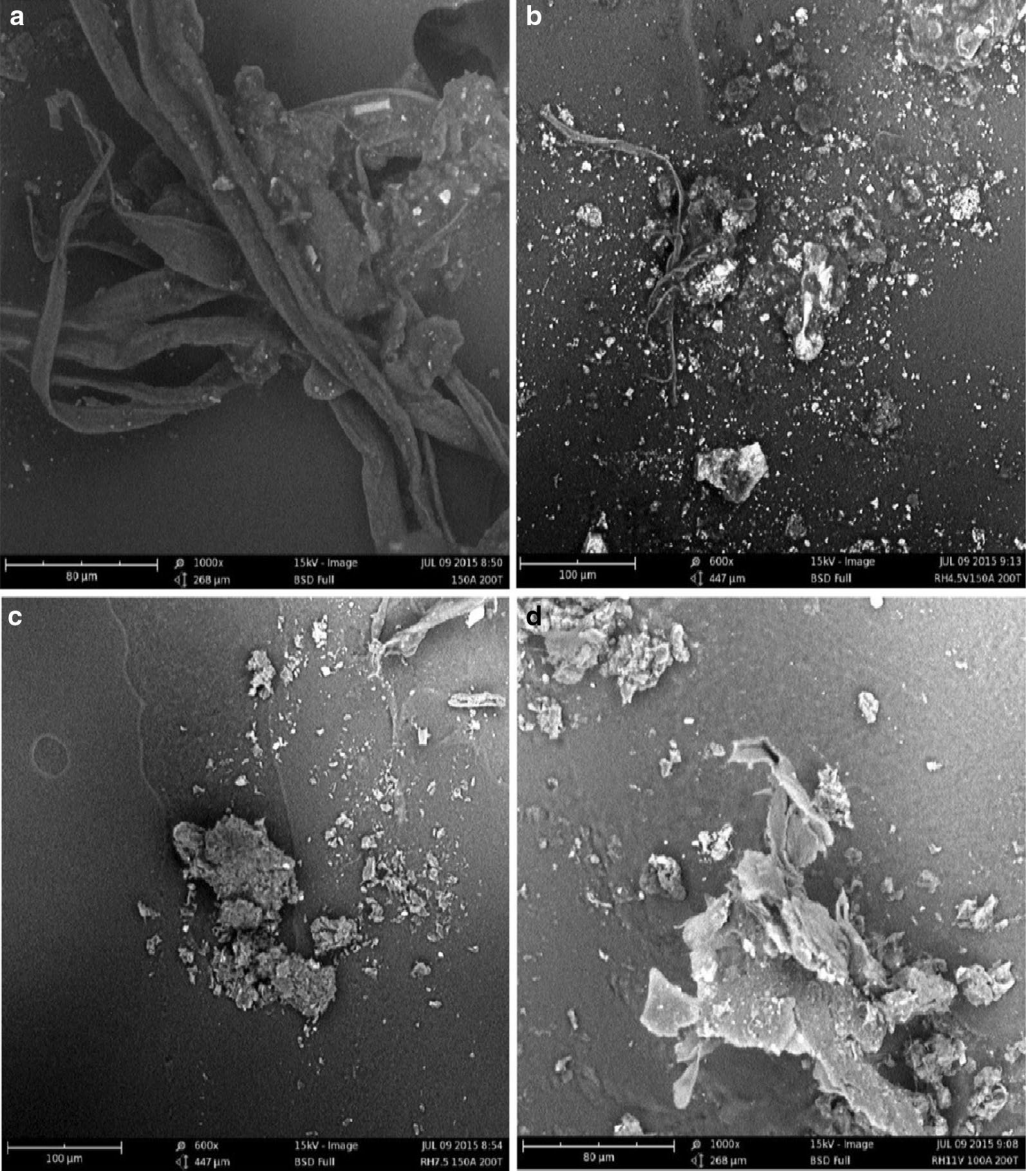
SEM images of fullerenes solid extracted from carbon soot collected at arc discharge system operating condition 150 A, 200 Torr chamber pressure. **a** no resistive heating, **b** 4.5 V resistive heating, **c** 7.5 V resistive heating, **d** 11 V resistive heating

Scanning electron microscopy (SEM) micrographs of fullerenes solid extracted from carbon soot collected from arc discharge system operating conditions of 150 A discharge current and 200 Torr chamber pressure with no resistive heating, 4.5 V resistive heating, 7.5 V resistive heating and 11 V resistive heating of one of the electrodes are as shown in Fig. 4a–d respectively.

#### Ultraviolet visible (UV–Vis) spectroscopy of fullerenes

**Fig. 5:**
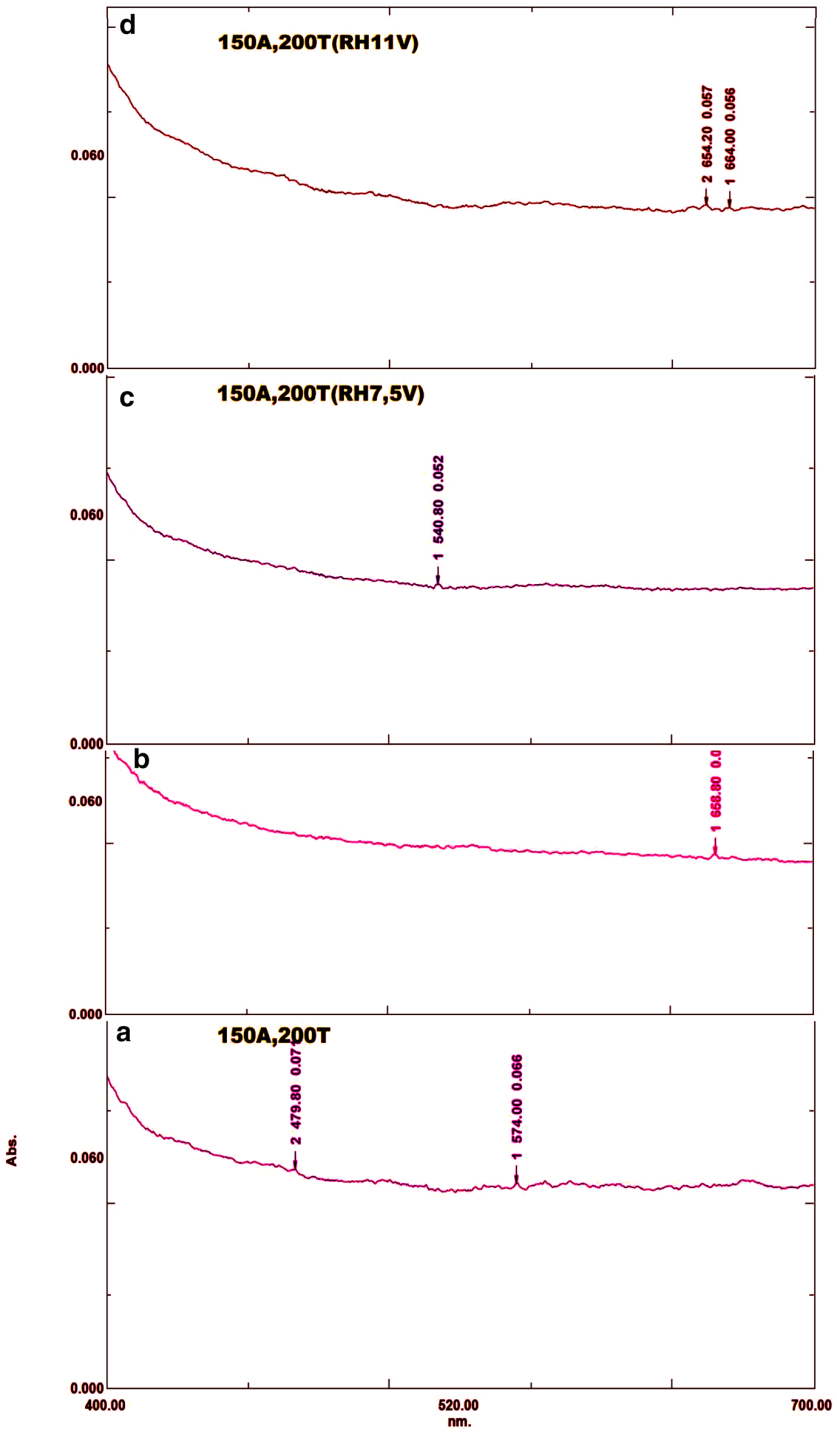
UV–Vis spectra of fullerenes solid extracted from carbon soot collected at different arc discharge operating conditions of discharge current, chamber pressure and resistive heating (RH) voltage. **a** 150 A, 200 Torr and no resistive heating, **b** 150 A, 200 Torr and RH 4.5 V, **c** 150 A, 200 Torr and RH 7.5 V, **d** 150 A, 200 Torr and RH 11 V

Figure 5a shows the UV–Vis Spectra of fullerenes solid extracted from carbon soot of collected at discharge current of 150 A and 200 Torr of chamber pressure with absorption peaks at 479.80 and 574.00 nm. Figure 5b–d below shows the UV–Vis Spectra of fullerenes solid extracted from carbon soot collected at 150 A of discharge current, 200 Torr of chamber pressure and when of of the electrodes was resistively heated at 4.5, 7.5 and 11 V respectively. Figure 5b shows UV–Vis spectra with absorption peak at 658.80 nm, Fig. 5c, shows the UV–Vis Spectra of UV–Vis spectra with absorption peak at 540.80 nm and Fig. 5d shows the UV–Vis Spectra with absorption peaks at 654.20 and 664.00 nm.

## Discussion

From the Table 1, the mass of carbon soot collected after 2 min of arc discharge run when one of the electrodes was resistively heating at 4.5, 7.5 and 11 V were all above twice the mass collected when no resistive heating was carried out. The highest carbon soot was collected when one of the electrodes was resistive heated at 11 V where 110.00 mg of carbon soot was collected for 2 min of arc discharge run was about 2.52 times the mass of soot collected when there wasn’t resistive heating of one of the electrodes. It was observed that carbon soot yield generally increases with resistive heating of one of the electrodes as seen in Fig. 2. This is because with Increasing resistive heating voltage, the heating rate of the electrode increases in accordance with ohmic heating principles. This will increase the rate of vaporization of the electrode leading to more carbon soot yield.

From Table 1 and Fig. 2, fullerenes solid extracted from 250 mg of carbon soot collected from arc discharge operating condition of 150 A discharge current and 200 Torr chamber pressure was 6 % which was within the expected 5–10 % of the total mass (Hare et al. 1991). Where one of the electrodes was resistively heated at 4.5, 7.5 and 11 V, best fullerenes yield of 4 % was extracted from carbon soot collected when one of the electrodes was resistively heated at 11 V. The fullerenes yield when one of the electrodes was heated resistively at 7.5 V (2.8 %) was less than the yield at 4.5 V (3.6 %). Although the reason for this could not be ascertained but it may be because heating the electrode at 7.5 V could have lead to the graphite rod vapourizing at temperature that does not support fullerenes formation. At 11 V of resistively heating one of the electrodes, more carbon rod vaporized as expected but the temperature would have been quite high enough to support the formation of fullerenes. This may be why the fullerenes yield of carbon soot collected under this arc discharge operating condition was higher.

The mass of fullerenes solid present in carbon soot collected per 2 min of arc discharge run when one of the electrodes was resistively heated were higher compared to when resistive heating of one of the electrodes was not carried out as seen on Fig. 3. This is as a result of the increase graphite vaporization due to ohmic heating giving rise to increase fullerenes formation. The highest fullerenes solid present in the carbon soot collected for 2 min of arc discharge run when one of the electrodes was resistively heated at 11 V was 4.4 mg. This was 67.9 % higher than when no resistive heating was carried out (at 150 A discharge current, 200 Torr chamber pressure).

SEM micrograph of fullerenes extract obtained from carbon soot of all arc discharge system operating conditions revealed some tubule-like whiskers, with length falling within the 40–15 mm corresponding to those of fullerenes whiskers as shown on Fig. 4 (Kingsuk et al. 1994).

Since the fullerenes solid extracted from carbon soot collected during the discharge system operating condition of 150 A of discharge current and 200 Torr of chamber pressure recorded absorption peak at 574 nm which correspond to the weak absorption band for C84 of ~574 nm (Kikuchi et al. 1991). The UV–Vis Spectra of fullerenes solid extracted from carbon soot obtained when one of the electrodes was resistively heated at 7.5 and 11 V recorded absorption peaks at 540.80 and 664.00 nm which corresponds to the weak absorption peaks of C_60_ and C_70_ respectively (Hare et al. 1991). The characteristic peak of the UV–Vis Spectra of fullerenes solid extracted from carbon soot obtained when one of the electrodes was resistively heated at 4.5 V could not be identified. The difficulty in identifying some of the characteristic peaks of the UV–Vis Spectra of fullerenes solid could be because the individual constituents of fullerenes solid in this work were not separated.

## Conclusion

The mass of fullerenes solid extracted for every 2 min of arc discharge run when resistive heating and direct arc techniques were used simultaneously gave higher yields compared to when only the direct arc technique was used (at discharge current of 150 A and chamber pressure of 200 Torr). At instances where resistive heating and direct arc techniques were used simultaneously, fullerenes solid present in carbon soot collected for 2 min of arc discharge run gave maximum yield of 4.4 mg when one of the electrodes was resistively heated at 11 V. This was 1.679 times the fullerenes solid present in carbon soot collected for 2 min of arc discharge run when only the direct arc technique was used (without resistive heating.

All the analysis carried out on the fullerenes solid extracted indicated the presence of fullerenes. The SEM analysis carried out on fullerenes solid indicates the presences of fullerenes whiskers while the UV–Vis spectra for fullerenes solids showed characteristic peaks for C_60_, C_70_ and C_84_.

## References

[CR1] Caraman MG, Lazar M, Stamate IL (2006). Arc discharge installation for fullerene production. Rom J Phys.

[CR2] Charlse ML, Chia-Chun C (1994). Preparation of fullerenes and fullerene-based materials. Solid State Phys.

[CR3] Churilov GN (2013). Synthesis of fullerenes and other nanomaterials in arc discharge. Fuller Nanotub Carbon Nanostruct.

[CR4] Hare JP, Kroto HW, Taylor R (1991). Preparation and ‘UV/visible spectra of fullerenes C_60_ and C_70_. Chem Phys Lett.

[CR5] Kikuchi K, Nakahara N, Honda M, Susuki S, Saito K, Shiromaru H, Yamauchi K, Ikemoto I, Kuramochi T, Hino S, Achiba Y (1991). Seperation, detection, and UV/visible absorption spectra of fullerenes; C_76_, C_78_ and C_84_. Chem Lett.

[CR6] Kingsuk M, Kalaga MK, Maheshwar S (1994). Fullerenes: C_60_ from camphor—a novel approach. Curr Sci India.

[CR7] Krolow MZ, Hartwig CA, Link GC, Raubach CW, Pereira JS, Picoloto RS, Gonçalves MR, Gonçalves NL, Mesko MF, Avellaneda C (2013). Synthesis and characterisation of carbon nanocomposites. NanoCarbon.

[CR8] Nirupam A, Navid BS, Jaime P-T (2015). Carbon nanomaterials for advanced energy systems.

[CR9] Yuming Z, Guang C (2014). C_60_ fullerene amphiphiles as supramolecular building blocks for organized and well-defined nanoscale objects. Struct Bond.

